# Changing Epidemiology of Clinical Isolates of *Candida* Species during the Coronavirus Disease 2019 Pandemic: Data Analysis from a Korean Tertiary Care Hospital for 6 Years (2017–2022)

**DOI:** 10.3390/jof10030193

**Published:** 2024-03-02

**Authors:** Eun Jeong Won, Heungsup Sung, Mi-Na Kim

**Affiliations:** Department of Laboratory Medicine, Asan Medical Center, University of Ulsan College of Medicine, Seoul 05505, Republic of Korea; sung@amc.seoul.kr (H.S.); mnkim@amc.seoul.kr (M.-N.K.)

**Keywords:** COVID-19 pandemic, *Candida* species, candidemia, *C. parapsilosis*

## Abstract

This study assessed the changes in *Candida* species distribution and antifungal susceptibility patterns during the coronavirus disease 2019 (COVID-19) pandemic compared with a pre-pandemic period in Korea. We retrospectively investigated the specimen, species type, and antifungal susceptibility of *Candida* isolates obtained between 2016 and 2022. Data between two periods were compared: 2016–2019 (pre-pandemic) and 2020–2022 (pandemic). We included 11,396 clinical isolates of *Candida* species (5137 isolates in the pre-pandemic and 6259 isolates in the pandemic). The most prevalent species was *Candida albicans* (50.4%), followed by *Candida glabrata* (22.7%), *Candida tropicalis* (12.5%), and *Candida parapsilosis* complex (12.5%). Their ranks were unchanged; however, their relative isolation ratios varied during the pandemic, exhibiting differences ranging from 0.4 to 2.5 across species. The incidence of candidemia increased during the pandemic (average 1.79 episodes per 10,000 patient days) compared with pre-pandemic levels (average 1.45 episodes per 10,000 patient days) in both intensive-care-unit (ICU) and non-ICU patients. Additionally, *C. parapsilosis* complex candidemia increased by 1.6-fold during the pandemic. During the pandemic, *C. albicans* and *C. tropicalis* candidemia significantly increased by 1.5- and 1.4-fold in ICU patients. In contrast, *C. parapsilosis* complex candidemia surged 2.1-fold in non-ICU patients. These species exhibited reduced resistance to fluconazole, voriconazole, caspofungin, and micafungin in the pandemic compared with the pre-pandemic. This study underscores the heightened incidence of *Candida*-related infections during the COVID-19 pandemic and emphasizes the importance of ongoing surveillance of *Candida* species epidemiology beyond the pandemic’s scope.

## 1. Introduction

In March 2020, the World Health Organization declared Coronavirus Disease 2019 (COVID-19), a respiratory infection caused by severe acute respiratory syndrome coronavirus 2 (SARS-CoV-2), a global pandemic, with the final declaration of the end to COVID-19 as a Global Health Emergency made on 4 May 2023 [1,2]. The emergence of COVID-19 caused an unprecedented public health crisis and brought new healthcare challenges, including the development of fungal coinfections [3]. Invasive candidiasis, comprising deep-seated tissue candidiasis and candidemia, is the most common fungal disease in hospitalized patients in developed countries [4]. The epidemiology of *Candida* infection has changed over the past several decades and in different geographical regions [5]. These changes have occurred because of different predisposing factors in patients, more aggressive therapy practices (e.g., chemotherapy, transplantation, and intensive care use), antifungal use, and other factors, as well as the recent COVID-19 pandemic [6]. Candidemia incidence during the pandemic was five times higher than that before the pandemic, and a 10-fold increase in candidemia frequency was observed in hospitalized patients with COVID-19 receiving corticosteroids compared with patients without COVID-19 [7,8,9]. This trend has been partly explained by immune paralysis or enhanced intestinal translocation in the patients [10] or a switch of microbiota toward *Candida* species [11], which then proceed to dominate the viral species. Recent data from the Korean Global Antimicrobial Resistance Surveillance System (Kor-GLASS) showed that *Candida* species ranked fourth among all target blood pathogens and second among all hospital-onset blood pathogens [12]. However, longitudinal epidemiological studies elucidating the changes in the distribution and antimicrobial susceptibility of *Candida* species related to the COVID-19 pandemic are still lacking. Thus, this study aimed to assess the changes in the distribution and antifungal susceptibility patterns of *Candida* species during the COVID-19 pandemic compared with a pre-pandemic period in Korea.

## 2. Materials and Methods

We collected the specimen type and antifungal susceptibility data of all clinical isolates of *Candida* species from the laboratory information system including the electronic medical record system (EMR) of a single-tertiary medical center in Korea containing 2732 beds from January 2017 to December 2022. We collected the data of all isolates of *Candida* species in the MycoBank database with a *Candida* anamorph name or previously classified as *Candida* [12]. A positive blood culture for *Candida* species in a person residing within our surveillance area was defined as a candidemia case. A *Candida* blood culture collected more than 30 days after the initial positive culture in the same person was considered a new case. The incidence of candidemia was defined as the number of cases of candidemia per 10,000 patient days (10,000 PD) [12]. Study periods were divided into two parts: January 2017 to December 2019 (COVID-19 pre-pandemic) and January 2020 to December 2022 (COVID-19 pandemic). Species identifications were performed using the Vitek 2 yeast identification card (Vitek 2 Yeast ID; bioMérieux, Marcy l’Etoile, France) according to the manufacturer’s instructions or matrix-assisted laser desorption/ionization time-of-flight mass spectrometry (Bruker Biotyper; Bruker Daltonics GmbH, Bremen, Germany). In vitro antifungal testing results for susceptibility to fluconazole, voriconazole, caspofungin, and micafungin were performed by Vitek 2 (bioMérieux, Marcy l’Etoile, France) and were interpreted according to the guidelines in the CLSI document M60 ED1 using species-specific clinical breakpoints (CBPs) [13]. This study was approved by the Institutional Review Board (IRB) of Asan Medical Center (approval no. 2023-0467). The IRB waived the requirement for informed consent because of the retrospective nature of this study.

## 3. Results

### 3.1. Overall Distribution of Candida Species Obtained from Clinical Samples during the Two Study Periods

Table 1 shows the overall distribution of *Candida* species obtained from clinical samples during the two study periods (2017–2022). A total of 11,396 clinical isolates of *Candida* species were included, with 5137 isolated in the pre-pandemic period and 6259 isolated in the pandemic period. The number of clinical isolates of *Candida* species increased annually from 17.29 per 10,000 PD in 2017 to 23.98 per 10,000 PD in 2022. The most prevalent species was *Candida albicans* (n = 5743, 50.4%), followed by *Candida glabrata* (n = 2588, 22.7%), *Candida tropicalis* (n = 1419, 12.5%), *Candida parapsilosis* complex (n = 893, 12.5%), *Clavispora lusitaniae* (n = 187, 1.6%), *Candida auris* (n = 181, 1.6%), *Pichia kudriavzevii* (n = 163, 1.4%), and other species (n = 212, 1.8%). The rank of the first four common *Candida* species was the same every year. During the pandemic period, these four *Candida* species were isolated 1.2- to 1.4-fold more than in the pre-pandemic period. The relative ratio of isolation (pandemic/pre-pandemic) was different for each *Candida* species, ranging from 0.4 to 2.5 (Supplementary Figure S1). Other than four common *Candida* species, *C. auris* was frequently isolated from clinical isolates. However, more than 90% of *C. auris* isolates were recovered from ear discharge and there was no bloodstream infection outbreak. The isolation of *C. auris* was decreased during the pandemic compared with the pre-pandemic.

### 3.2. Dynamics of Candidemia Incidence Pre-Pandemic and Pandemic Periods

Table 2 summarizes the incidence of candidemia during the study periods. The incidence of candidemia increased during the pandemic (average 1.79 per 10,000 PD) compared with the pre-pandemic period (average 1.45 per 10,000 PD). Candidemia cases caused by *C. albicans*, *C. glabrata*, *C. tropicalis*, and *C. parapsilosis* complex during the pre-pandemic/pandemic were 0.49/0.60, 0.46/0.57, 0.27/0.34, and 0.12/0.18 per 10,000 PD, respectively. The incidence of candidemia caused by *C. parapsilosis* complex significantly increased 1.6-fold during the pandemic compared with the pre-pandemic.

### 3.3. Distribution of Candidemia Incidence between Intensive-Care-Unit (ICU) and Non-ICU Patients

Analyzing the distribution of candidemia incidence between intensive-care-unit (ICU) and non-ICU patients, a relative increment in candidemia cases was observed in the pandemic period, with a 1.1-fold increase in ICU patients and a 1.2-fold increase in non-ICU patients (Table 3). In ICU patients, candidemia cases caused by *C. albicans* and *C. tropicalis* were significantly increased during the pandemic (1.5- and 1.4-fold more compared with the pre-pandemic, respectively). In contrast, candidemia cases caused by *C. glabrata* and *C. parapsilosis* complex were slightly decreased during the pandemic (0.9- and 0.7-fold lower compared with the pre-pandemic, respectively). In non-ICU patients, candidemia cases caused by the four common *Candida* species and *P. kudriavzevii* increased during the pandemic, ranging from 1.1- to 2.1-fold compared with the pre-pandemic period.

### 3.4. The Effect of the COVID-19 Pandemic on Antifungal Susceptibility Patterns

Finally, we assessed the effect of the COVID-19 pandemic on antifungal susceptibility patterns (Table 4). By applying species-specific CBPs, non-susceptibility to fluconazole, voriconazole, caspofungin, and micafungin was observed to be 42.3%, 5.7%, 4.6%, and 2.8% in the pre-pandemic period and 35.8%, 0.0%, 3.6%, and 1.2% in the pandemic period, respectively. During the pre-pandemic period, 8.1%, 2.5%, 0.0%, and 7.0% of *C. albicans*, *C. glabrata*, *C. tropicalis*, and *C. parapsilosis* complex isolates, respectively, showed resistance to fluconazole. However, isolates of these species did not exhibit resistance to fluconazole during the pandemic. Additionally, resistance to caspofungin was observed to be 0.9%/0.6% for *C. albicans* and 10.0%/5.7% for *C. glabrata* during the pre-pandemic/pandemic periods, respectively. In contrast, resistance to micafungin was 0.9%/0.6% for *C. albicans* and 0.0%/0.6% for *C. glabrata* during the pre-pandemic/pandemic periods, respectively.

## 4. Discussion

The recent emergence of COVID-19 has resulted in an unprecedented public health crisis and brought new healthcare challenges, including the development of fungal coinfections such as candidemia [3]. The increase in the incidence of candidemia during the pandemic reported in our study is consistent with the results of previous studies conducted in different geographic regions [3,7,15]. A Brazilian single-center study observed a 5.7-fold increment in candidemia during the COVID-19 pandemic, although their incidence of candidemia had been stable over the past 18 years [7]. As shown in Kor-GLASS data obtained from nine collection centers, the average incidence of candidemia was 1.45 cases per 10,000 PD in 2020 and 1.72 cases per 10,000 PD in 2021 [12]. This was partly in line with our single center data showing 1.79 episodes per 10,000 PD during the pandemic. This increment in the incidence of candidemia may be explained by two factors: an increase in the absolute number of episodes of candidemia (404 episodes in pre-pandemic vs. 484 episodes in pandemic) and a decrease in the number of PDs during the pandemic (average PDs per year, 931,086 PDs in pre-pandemic vs. 902,741 PDs in pandemic). Previous research has suggested that COVID-19 could easily contribute to the development of candidemia due to common risk factors between the diseases, such as the use of antibiotics, corticosteroids, venous catheters, and dialysis; furthermore, mucosal barrier disruption caused by COVID-19 could facilitate the translocation of *Candida* species from the gut to the bloodstream [16]. Moreover, patients with severe COVID-19 are predisposed to acquire secondary fungal infections such as COVID-19-associated candidemia and are associated with poor clinical outcomes despite receiving antifungal treatment [17]. Our study highlights the importance of cautiously monitoring invasive candidiasis in the post-pandemic era.

Given that each *Candida* species has a unique virulence potential, antifungal susceptibility pattern, and clinical characteristics [18], the changing epidemiology of candidemia may have different implications for the management of—and mortality due to—ICU-associated candidemia depending on the *Candida* species. In this study, in ICU patients, candidemia caused by *C. albicans* and *C. tropicalis* was significantly increased by 1.5- and 1.4-fold during the pandemic. This is a cause for concern, considering that the mortality rates of *C. albicans* and *C. tropicalis* candidemia in ICU patients are approximately 2-fold higher than those in non-ICU patients [19]. Meanwhile, *C. parapsilosis* complex candidemia surged 2.1-fold in non-ICU patients and 1.6-fold overall during the pandemic. Moreover, candidemia due to *C. parapsilosis* was 2-fold more persistent than that caused by other common *Candida* species, and this persistence was strengthened during the pandemic. Previously, the clinical implication of *C. parapsilosis* was relatively less recognized than those of other common *Candida* species. Recently, candidemia caused by fluconazole-non-susceptible *C. parapsilosis* isolates has been increasingly reported worldwide, and outbreaks of fluconazole-resistant isolates have been reported from several countries during the pandemic [20,21,22,23,24]. Besides sporadic infections, *C. parapsilosis* is well known to cause nosocomial outbreaks through direct and indirect contact via the hands of healthcare workers and through contaminated patient care equipment. It is likely that the limited availability of personal protective equipment and the congestion in hospital units during the pandemic created a permissive environment for the emergence of such outbreaks [24,25,26]. The persistence of such infections has led to changes in clinical practice and the replacement of fluconazole with echinocandins [22,27,28]. Notably, a recent Korean study proposed that the long-term clonal transmission of *C. parapsilosis* isolates in Korean hospitals may be linked to specific sinking phenotypes [28]. Their microbiological characteristics could make them prone to azole-resistant mutation, causing them to persist for a long time in the environment, where they may cause bloodstream infections in vulnerable patients with indwelling catheters or azole exposure. In our study, antifungal resistance remained low and clonal spreading was not a big concern during the pandemic, similar to previous reports [12,29]. However, it is necessary to monitor the changing epidemiology of indigenous *C. parapsilosis* in Korea, considering their persistent nature and potential to become antifungal-resistant.

In this study, we found that *C. auris* was frequently isolated from ear discharge, rather causing a bloodstream infection or outbreak, and the isolation of *C. auris* was decreased during the pandemic. This might be explained by characteristics of the East Asian clade (clade II), which were inherently different from other clades [19,30,31]. A recent study suggested that several factors such as reduced thermal tolerance at 42 °C, reduced virulence in the *G. mellonella* model, and a reduced competitive growth ability compared to non-clade II isolates might contribute to the decreased colonization by clade II *C. auris* in hospital settings and at various body sites and to their absence in nosocomial transmissions [31]. However, further surveillance should be continued considering the recent emergence of six clade I isolates of *C. auris* in a Korean hospital [31], harboring the potential to be a nosocomial cluster in Korea.

## 5. Conclusions

Infections due to *Candida* species increased during the COVID-19 pandemic. A rise in candidemia cases caused by *C. parapsilosis* was observed in Korea during the pandemic. Such changes in the epidemiology of *Candida* species should be continuously monitored in the post-pandemic period. This research underscores the heightened incidence of *Candida*-related infections during the COVID-19 pandemic and emphasizes the importance of ongoing surveillance of *Candida* species epidemiology beyond the pandemic’s scope.

## Figures and Tables

**Table 1:** Distribution of clinical isolates of *Candida* species at a single-tertiary medical center in Korea from 2017 to 2022.

Species	Subtotal No. (%)	Number of Clinical Isolates of *Candida* Species Obtained from 2017 to 2022								Fold (Pandemic/Pre-Pandemic)
		2017	2018	2019	2020	2021	2022	Pre-Pandemic ^a^	Pandemic ^b^	
*Candida albicans*	5743 (50.4)	807	902	946	992	1006	1090	2655	3088	1.2
*Candida glabrata*	2588 (22.7)	353	368	395	455	538	479	1116	1472	1.3
*Candida tropicalis*	1419 (12.5)	212	185	231	272	242	277	628	791	1.3
*Candida parapsilosis* complex ^c^	893 (7.8)	114	132	133	138	181	195	379	514	1.4
*Clavispora lusitaniae*	187 (1.6)	16	25	28	34	28	56	69	118	1.7
*Candida auris*	181 (1.6)	43	27	31	31	21	28	101	80	0.8
*Pichia kudriavzevii*	163 (1.4)	26	33	23	25	34	22	82	81	1.0
*Meyerozyma guilliermondii*	52 (0.5)	11	9	12	7	9	4	32	20	0.6
*Kluyveromyces marxianus*	38 (0.3)	1	4	9	9	9	6	14	24	1.7
*Cyberlindnera jadinii*	21 (0.2)	2	8	1	5	3	2	11	10	0.9
*Trichomonascus ciferrii*	10 (0.1)		4	2		3	1	6	4	0.7
*Candida dubliniensis*	10 (0.1)	1	4	2	1		2	7	3	0.4
*Candida intermedia*	7 (0.1)			2	2	1	2	2	5	2.5
Other *Candida* species ^d^	12 (0.1)	1	2	5	1	2	1	8	4	0.5
*Candida,* not *albicans*	72 (0.6)	13	8	6	24	6	15	27	45	1.7
Total	11,396 (100)	1600	1711	1826	1996	2083	2180	5137	6259	1.2
Incidence/10,000 patient-days		17.29	18.20	19.69	22.69	22.66	23.98	18.39	23.11	1.3

^a^ COVID-19 pre-pandemic period was designated from January 2017 to December 2019; ^b^ COVID-19 pandemic period was designated from January 2020 to December 2022; ^c^ *Candida parapsilosis* complex included *Candida parapsilosis* sensu stricto, *Candida orthopsilosis*, and *Candida metapsilosis* [14]; ^d^ Other *Candida* species included *Candida haemulonii* (n = 3), *Vanrija humicola* (n = 3), *Diutina catenulata* (n = 2), *Yarrowia lipolytica* (n = 1), *Diutina rugosa* (n = 1), *Candida magnoliae* (n = 1), unknown *Candida* species (n = 1).

**Table 2:** Incidence of candidemia during the pre-pandemic and the COVID-19 pandemic in this study.

Incidence/10,000 Patient-Days	Incidence of Candidemia Patients (Episodes/10,000 Patient-Days)								Fold
	2017	2018	2019	2020	2021	2022	Pre-Pandemic ^a^	Pandemic ^b^	(Pandemic/Pre-Pandemic)
*Candida albicans*	0.58	0.45	0.45	0.53	0.63	0.63	0.49	0.60	1.2
*Candida glabrata*	0.36	0.57	0.44	0.6	0.58	0.54	0.46	0.57	1.2
*Candida tropicalis*	0.28	0.22	0.3	0.36	0.39	0.27	0.27	0.34	1.3
*Candida parapsilosis* complex ^c^	0.13	0.11	0.12	0.14	0.2	0.22	0.12	0.18	1.6
*Pichia kudriavzevii*	0.05	0.04	0.03	0.03	0.03	0.05	0.04	0.04	1.0
Total candidemia	1.44	1.48	1.42	1.7	1.88	1.77	1.45	1.79	1.2

^a^ COVID-19 pre-pandemic period was designated from January 2017 to December 2019; ^b^ COVID-19 pandemic period was designated from January 2020 to December 2022; ^c^ *Candida parapsilosis* complex included *Candida parapsilosis* sensu stricto, *Candida orthopsilosis*, and *Candida metapsilosis* [14].

**Table 3:** Distribution of *Candida* species isolated from blood cultures of intensive-care-unit (ICU) patients or non-ICU patients and relative ratio of candidemia incidence between the pre-pandemic and COVID-19 pandemic periods.

Species	Number of Candidemia Patients				Relative Ratio of Candidemia Incidence (Pandemic/Pre-Pandemic)	
	ICU		Non-ICU			
	Pre-Pandemic	Pandemic	Pre-Pandemic	Pandemic	ICU	non-ICU
*Candida albicans*	31	47	107	115	1.5	1.1
*Candida glabrata*	39	35	89	120	0.9	1.3
*Candida parapsilosis* complex ^a^	13	9	20	41	0.7	2.1
*Candida tropicalis*	18	26	57	67	1.4	1.2
*Pichia kudriavzevii*	4	2	8	9	0.5	1.1
*Meyerozyma guilliermondii*	0	0	6	1	N/A	0.2
Other *Candida* species ^b^	5	6	7	6	1.2	0.9
Total	110	125	294	359	1.1	1.2

Abbreviations: N/A, not available; ICU, intensive care unit. ^a^ *Candida parapsilosis* complex included *Candida parapsilosis* sensu stricto, *Candida orthopsilosis*, and *Candida metapsilosis* [14]; ^b^ Other *Candida* species included *Clavispora lusitaniae* (n = 17), *Kluyveromyces marxianus* (n = 3), *Cyberlindnera jadinii* (n = 1), *Candida magnoliae* (n = 1), *Candida dubliniensis* (n = 1), unknown *Candida* species (n = 1).

**Table 4:** Antifungal susceptibilities of the six common *Candida* species obtained from blood cultures according to the pre-pandemic and COVID-19 pandemic.

Antifungal Agent ^a^		Pre-Pandemic			Pandemic			Total			
/Species		No.	%R	%SDD/I	No.	%R	%SDD/I	No.	%R	%SDD/I	%NS
Fluconazole	*Candida albicans*	111	8.1	1.8	165	0.0	0.6	276	3.3	1.1	4.4
	*Candida tropicalis*	62	0.0	0.0	93	0.0	1.1	155	0.0	0.7	0.7
	*Candida glabrata*	120	2.5	97.5	159	0.0	100.0	279	1.1	98.9	100.0
	*Candida parapsilosis* complex ^b^	43	7.0	2.3	62	0.0	1.6	105	2.9	1.9	4.8
	*Pichia kudriavzevii*	12	100.0	0.0	15	100.0	0.0	27	100.0	0.0	100.0
	Total	348	7.8	34.5	494	3.0	32.8	842	5.0	33.5	38.5
Voriconazole	*Candida albicans*	111	7.2	1.8	165	0.0	0.0	276	2.9	0.7	3.6
	*Candida tropicalis*	62	0.0	0.0	93	0.0	0.0	155	0.0	0.0	0.0
	*Candida parapsilosis* complex ^b^	43	2.3	2.3	62	0.0	0.0	105	1.0	1.0	2.0
	*Pichia kudriavzevii*	12	0.0	8.3	15	0.0	0.0	27	0.0	3.7	3.7
	Total	228	3.9	1.8	335	0.0	0.0	563	1.6	0.7	2.3
Caspofungin	*Candida albicans*	111	0.9	0.0	165	0.6	0.0	276	0.7	0.0	0.7
	*Candida tropicalis*	62	0.0	0.0	93	0.0	0.0	155	0.0	0.0	0.0
	*Candida glabrata*	120	10.0	1.7	159	5.7	4.4	279	7.5	3.2	10.7
	*Candida parapsilosis* complex ^b^	43	0.0	0.0	62	0.0	0.0	105	0.0	0.0	0.0
	*Meyerozyma guilliermondii*	5	0.0	0.0	1	0.0	0.0	6	0.0	0.0	0.0
	*Pichia kudriavzevii*	12	8.3	0.0	15	6.7	0.0	27	7.4	0.0	7.4
	Total	353	4.0	0.6	495	2.2	1.4	848	2.9	1.1	4.0
Micafungin	*Candida albicans*	111	0.9	0.0	165	0.6	0.0	276	0.7	0.0	0.7
	*Candida tropicalis*	62	0.0	0.0	93	0.0	0.0	155	0.0	0.0	0.0
	*Candida glabrata*	120	0.0	6.7	159	0.6	1.9	279	0.4	3.9	4.3
	*Candida parapsilosis* complex ^b^	43	0.0	0.0	62	0.0	0.0	105	0.0	0.0	0.0
	*Meyerozyma guilliermondii*	5	0.0	0.0	1	0.0	0.0	6	0.0	0.0	0.0
	*Pichia kudriavzevii*	12	0.0	8.3	15	6.7	0.0	27	3.7	3.7	7.4
	Total	353	0.3	2.5	495	0.6	0.6	848	0.5	1.4	1.9

Abbreviations: R, resistant; I, intermediate; SDD, susceptible-dose-dependent; NS, non-susceptible. ^a^ Percentages of isolates categorized by the Clinical and Laboratory Standards Institute (CLSI M60) species-specific clinical breakpoints [13]. ^b^ *Candida parapsilosis* complex included *Candida parapsilosis* sensu stricto, *Candida orthopsilosis*, and *Candida metapsilosis* [14].

## Data Availability

Data are contained within the article.

## Supplementary Materials

The following supporting information can be downloaded at https://www.mdpi.com/article/10.3390/jof10030193/s1. Figure S1: Relative isolation ratio of *Candida* species obtained from clinical samples from the pandemic to pre-pandemic era.
