# Supplementary material for: Changing Epidemiology of Clinical Isolates of Candida Species during the Coronavirus Disease 2019 Pandemic: Data Analysis from a Korean Tertiary Care Hospital for 6 Years (2017–2022)
Source: J Fungi (Basel). 2024 Mar 2;10(3):193. doi: 10.3390/jof10030193 (PMC10971515; doi:10.3390/jof10030193)
Supplement: Supplementary file 1 [file jof-10-00193-s001.zip › jof-2783190-supplementary.pdf]

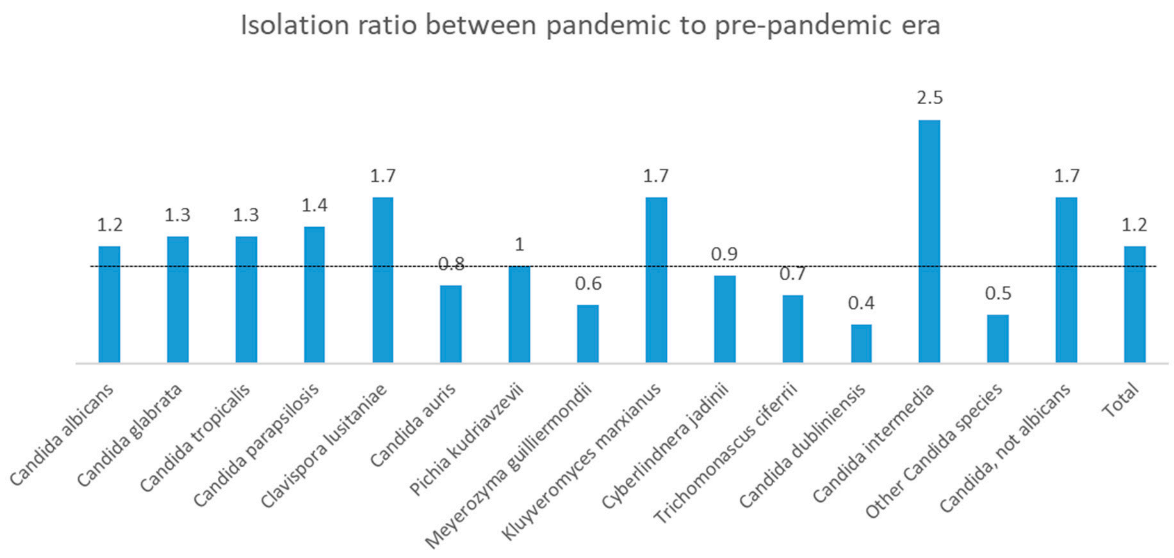

Supplemental Figure S1. Relative isolation ratio of *Candida* species obtained from clinical samples between pandemic to pre-pandemic era.
